# Cell Membrane Sialome: Sialic Acids as Therapeutic Targets and Regulators of Drug Resistance in Human Cancer Management

**DOI:** 10.3390/cancers15205103

**Published:** 2023-10-22

**Authors:** Patrycja Jastrząb, Karolina Narejko, Halina Car, Przemyslaw Wielgat

**Affiliations:** 1 Department of Clinical Pharmacology, Medical University of Bialystok, Waszyngtona 15A, 15-274 Bialystok, Poland; patrycja.jastrzab@sd.umb.edu.pl (P.J.); knarejko1@student.umb.edu.pl (K.N.); halina.car@umb.edu.pl (H.C.); 2 Department of Experimental Pharmacology, Medical University of Bialystok, Szpitalna 37, 15-295 Bialystok, Poland

**Keywords:** sialic acid, sialome, cancer, chemoresistance, therapy

## Abstract

**Simple Summary:**

The aberrant sialylation of membrane glycocalyx plays a pivotal role in the regulation of malignant cell behavior and correlates with a worse prognosis and shorter overall survival for patients. The biological and physical properties of sialome determine the negative charge and high hydrophilicity of cell membranes and thereby regulate cell–cell and cell–extracellular matrix interactions. There is increasing evidence that sialic acids influence cellular susceptibility in therapeutic management. Here, we focus on the engagement of sialic acids in chemoresistance and the potential effects of drugs and potential therapeutic agents on sialome-related machinery in malignant cells.

**Abstract:**

A cellular sialome is a physiologically active and dynamically changing component of the cell membrane. Sialylation plays a crucial role in tumor progression, and alterations in cellular sialylation patterns have been described as modulators of chemotherapy effectiveness. However, the precise mechanisms through which altered sialylation contributes to drug resistance in cancer are not yet fully understood. This review focuses on the intricate interplay between sialylation and cancer treatment. It presents the role of sialic acids in modulating cell–cell interactions, the extracellular matrix (ECM), and the immunosuppressive processes within the context of cancer. The issue of drug resistance is also discussed, and the mechanisms that involve transporters, the tumor microenvironment, and metabolism are analyzed. The review explores drugs and therapeutic approaches that may induce modifications in sialylation processes with a primary focus on their impact on sialyltransferases or sialidases. Despite advancements in cellular glycobiology and glycoengineering, an interdisciplinary effort is required to decipher and comprehend the biological characteristics and consequences of altered sialylation. Additionally, understanding the modulatory role of sialoglycans in drug sensitivity is crucial to applying this knowledge in clinical practice for the benefit of cancer patients.

## 1. Introduction

**Figure 1:**
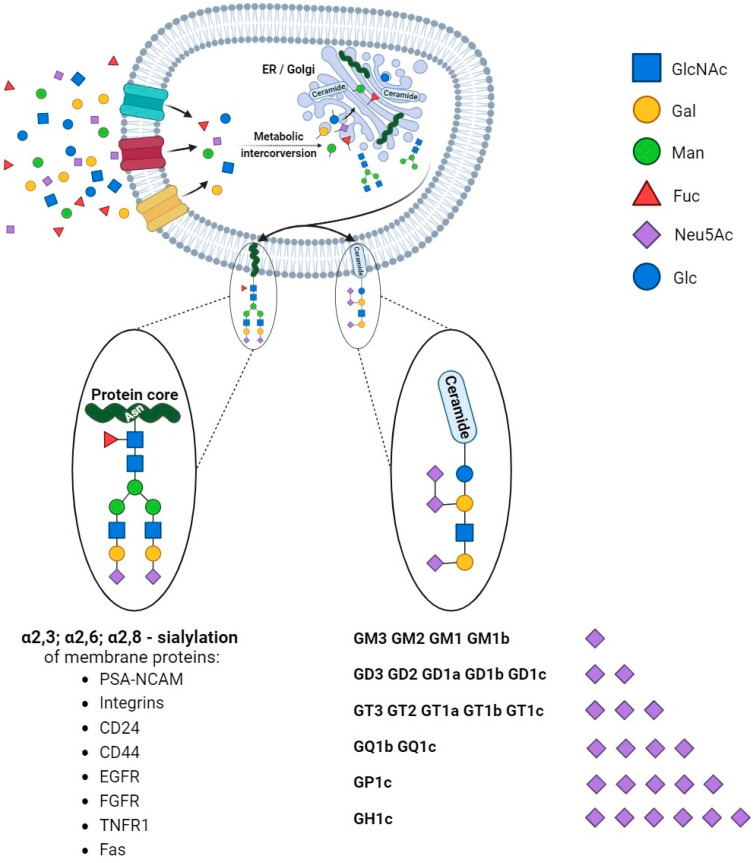
Synthesis and diversity of glycoproteins and glycolipids. The monosaccharide units are translocated into the cells and undergo modifications via phosphorylation and conjugation with nucleotides. The sequential assembly of glycoproteins and glycolipids is orchestrated through essential collaboration between the endoplasmic reticulum (ER) and the Golgi apparatus. Initial modifications occur in the ER, followed by subsequent refinements within the Golgi apparatus. In physiological and pathological conditions, proteins undergo sialylation, including those pivotal in cancer processes. Additionally, monosaccharides form intricate structures with lipids, exhibiting varying quantities of sialic acids (in the figure, ranked from the least to the most sialylated).

According to the concept by Cohen and Varki, cell membrane glycomolecules form a dense network characterized by a high grade of diversity and dynamic variability [1]. It makes these structures like a forest with complex “tree crowns” formed by glycans covalently conjugated to protein or a lipid core. The complex structure and function of the glycome depend on monosaccharide units and the stereospecific formation of glycosidic bonds that determine their combination and the spatial organization of glycans. Indeed, the glycoconjugate landscape is dynamically formed by glycome–metabolic enzyme and glycome–metabolic pathway modifying factors that result in specific glycosylation patterns and their critical role in molecular and cellular biology. As a result of metabolic conversion, the activated monosaccharide units of dietary or molecular carbohydrate recycling origin are assembled into complex sugar chains in the Golgi apparatus and attached to the protein or lipid core via glycosyltransferases [2,3] (Figure 1).

Structural studies on cellular glycomes have revealed the glycan epitopes of defined sequences featured in the wide expression of neuraminic acid derivatives routinely called sialic acids. The attachment of sialic acids to the non-reducing end of sugar chains is the last step of glycosylation and results in enhanced microheterogeneity that influences the physical and biochemical properties of glycoconjugates [4]. The specific location, ubiquity, and varied forms of these molecules make them one of the most versatile molecules participating in many physiological processes. They are of particular importance in the field of cell–cell and cell–extracellular matrix interactions that underlie biological recognition, cellular communication, adhesion, and migration. The high-specific sialoglycans have been shown to modulate the function of cell membrane receptors via alterations in dimerization, activation, and autophosphorylation that result in changes in cellular signaling and thereby regulate cell proliferation and survival [5]. The proper sialylation pattern is balanced via the activities of sialyltransferases and sialidases that play crucial roles in the maintenance of cellular homeostasis. Therefore, the ability to affect cellular processes makes sialylation a key player in human health in the fields of nephrology, hepatology, neurobiology, endocrinology, cardiovascular physiology, reproductive processes, and immune responses [6]. Advances in glycobiology and glycoengineering have revealed that the unbalanced synthesis and metabolism of sialoglycans is a pathological risk factor and predictive factor for cell and tissue dysfunction. Finally, aberrant sialylation has been described as a potential therapeutic target and modulator of conventional therapy efficiency. Molecular and clinical studies have confirmed the participation of sialic acids in human pathology; however, the role of sialoglycans in cancer deserves particular attention. Clinical and molecular investigations have confirmed alterations in the sialylation patterns of tumors of various histological types and origins. As shown, the presence of hypersialylated glycans is crucial for malignant cells’ behavior, particularly their activity in a tumor niche, cross-talk with surrounding normal tissue, and infiltrating immune cells [7]. This variation modulates immunosurveillance, making the cells more resistant to the immune response. Indeed, sialic-acid-related immune evasion is a hallmark of many malignancies closely associated with a poor prognosis [4]. Moreover, there is increasing evidence of the engagement of sialic acids in the drug resistance phenomenon in cancer management [8]. Although the modulatory effects of sialic acids on the drug action have been described, the exact mechanisms by which an altered sialylation pattern contributes to drug resistance in cancer are not yet fully understood. Both a poor therapeutic response and multidrug resistance are serious clinical problems; therefore, understanding the modulatory role of sialome in drug sensitivity seems to be important for the development and/or improvement of therapeutic efficiency. This review briefly focuses on the complex interplay between the cellular sialome and conventional therapies and their clinical importance in routine practice.

## 2. Sialic Acids in Cancer—Smart Players in a Complex Mechanism

### 2.1. Sialic Acids and the Regulation of Cellular Functions in Cancer

Sialome plays a complex and multifaceted role in the structure of cell membranes and the extracellular matrix; therefore, numerous alterations in this field are known to be critical for cancer progression. Similar to normal cells, the decoration of the cellular glycocalyx via sialic acids can impact cellular recognition, signaling, adhesion, molecular trafficking, and migration. However, cancer cells do not express the molecules on their membrane properly, and altered glycosylation and changes in glycoprotein expression are common features of tumorigenesis [9]. Among molecular variations, increased sialylation is a hallmark of several cancers, including breast, colorectal, lung, and brain tumors [7,10,11,12,13,14]. Many functions in cells and tissues are regulated via ionic flows, electric fields, and voltage gradients, so any aberrations that cause changes in charge will affect them [15]. Sialic acids are known to create a negative charge on the cell membrane that influences the hydrophilicity of the cell surface and regulates cellular adhesiveness. It is of particular importance in cell migration and determines the metastatic potential of malignant cells [7]. As shown, the overexpression of sialic-acid-terminated glycans leads to alterations in immune status and antibody functions that facilitate tumor formation and correlate with aggressiveness [7,16]. Among sialylated adhesion modulators, the polymers of alpha 2,8-linked sialic acid (polysialic acid, PSA) affect the adhesive properties of neuronal cell adhesion molecules (NCAMs) and perform a pivotal function in the dynamic neuronal generation in the embryonic and neonatal hippocampus [17,18,19,20]. The repulsive effects caused by PSA allow cells to migrate, as has been confirmed in various types of cancer [17,21,22]. Sialic-acid-dependent cell migration is potentiated via PSA in a hypoxic environment; however, it is still unknown how oxygen deficiency affects glycosylation and sialylation in cancer [23,24]. In addition to polysialylated NCAMs (PSA-NCAMs), the family of selectins is involved in adhesion processes during cancer progression. The interplay between selectins and their ligands, sialyl-Lewis-X (sLeX), promotes interactions between cancer cells and endothelial cells, leukocytes, or platelets and thereby plays an important role in metastasis via facilitating the extravasation of tumor cells from the bloodstream [25]. The meta-analysis by Liang et al. has shown that sialyl-Lewis-X overexpression is associated with cancer metastasis, disease recurrence, and overall survival in cancer patients [26]. The exact reasons for altered sialylation in cancer are still unknown. However, three probable mechanisms that may influence changes in sialome are indicated [14]. The first mechanism underlying hypersialylation may be associated with altered metabolic flux via cancer cells with the ability to modulate their metastatic potential. Metabolic reprogramming is one of the hallmarks of cancer, and the reprogrammed metabolism supports its development. Moreover, there are several reports dealing with the influence of metabolic flux on sialylation. The investigation by Almaraz et al. has indicated that the malignancy status becomes altered due to massive metabolic flux through the sialic acid pathway. As shown, the uptake of sialic acid precursors in tumor cells from the extracellular space results in a significant increase in glycoproteins sialylation [27].

### 2.2. Enzymatic Dysregulation of Sialylated Glycocalyx in Cancer

**Figure 2:**
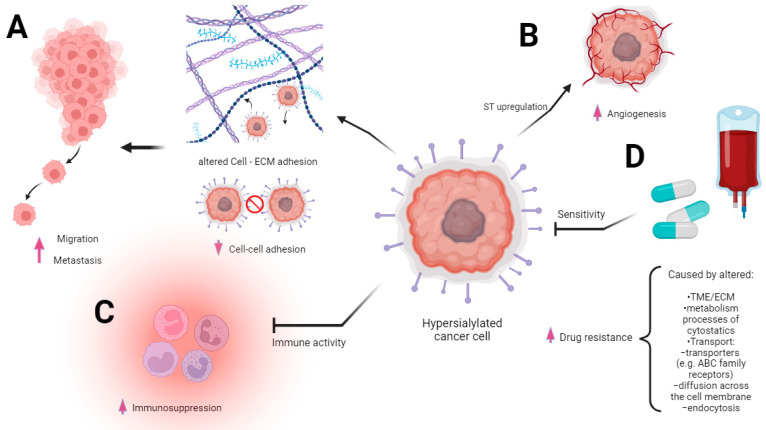
Multidirectional involvement of sialic acid overexpression in cancer progression. The figure underscores the multifaceted role of sialic acid overexpression in driving various aspects of cancer progression. The modified extracellular matrix (ECM) within the tumor microenvironment is altered via the heightened presence of sialic acid. This change in ECM composition fosters significant alterations in the migratory behavior of cancer cells (**A**). The heightened immunosuppression is mediated via sialic acid overexpression. Sialic acids and sialic acid binding receptors participate in evading immune surveillance and thereby facilitating cancer progression (**B**). The heightened level of sialic acids, contributing to the augmentation of angiogenesis processes, is intricately associated with alterations in ST expression. The sialic acids’ presence is intricately linked to an upsurge in angiogenic signals and fosters a conducive environment for tumor vascularization, culminating in enhanced vessel growth (**C**). Drug resistance may ensue from the coexistence of diverse mechanisms intricately linked to carcinogenesis with a high likelihood of multiple biochemically mediated pathways contributing to the emergence of resistance. Sialic acids’ overexpression is involved in altering drug influx and efflux mechanisms, a driving factor behind this acquired resistance (**D**).

Changes in the sialylation controlling machinery were found in several types of cancers and described as markers of sialome-related tumorigenesis. First, the aberrant sialylation processes in malignancies correspond to the altered expression and activity of endogenous sialidases in diverse cellular compartments. Among the four sialidases (NEU1, NEU2, NEU3, and NEU4), the enhanced expression of NEU3 was observed in malignant tissues, whereas the rest of these enzymes tended to be reduced, which led to the accumulation of sialoconjugates [14]. According to Miyagi et al., the altered sialidases of different subcellular localizations play pivotal roles in controlling transmembrane signaling, lysosomal catabolism, and the modulation of functional molecules involved in many biological processes [28]. Besides the sialidases, the family of glycosyltransferases is part of the complex mechanism underlying changes in glycosylation patterns that are closely related to human pathologies, including the pathology of cancer [29]. The transfer of sialic acid to the appropriate acceptors is catalyzed via the group of 20 sialyltransferases that are observed to be raised in many malignancies [30]. As a result, the overexpression of sialoglycans and their incorrect branching promote tumor migration and invasion through the influence of cancer cell angiogenesis, adhesion, and epithelial–mesenchymal transition (EMT) [31]. These effects are strongly correlated with the advanced stage of malignancy, high metastatic potential, and patients’ reduced overall survival [32,33,34] (Figure 2A,B).

**Figure 3:**
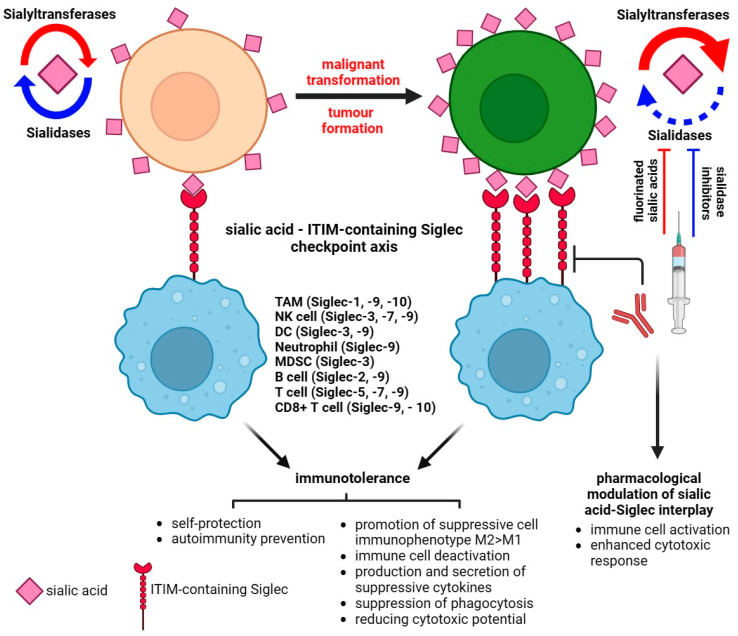
Sialic acid–Siglec checkpoint in immune control in health and cancer. The interplay between membrane sialoglycans and inhibitory Siglecs results in immunotolerance and the promotion of cancer progression. The pharmacological targeting of Siglec or sialome-modulatory machinery disturbs the immunosuppressive status of the tumor microenvironment and impairs the invasive potential of cancer.

The dysregulation of sialyltransferases depends on the mechanisms that involve genetic, epigenetic, transcriptional, and post-transcriptional regulations known to be developed in cancer and closely associated with the glycosylation pattern [32,35,36]. Finally, the biosynthesis and distribution of glycans in malignant cells are regulated via microenvironmental cues [37]. The tumor microenvironment (TME) is composed of immune system cells, cancer-associated fibroblasts (CAFs), that communicate with cancer cells and each other during tumor progression [38]. This is mentioned because even the extracellular matrix (ECM) consists of proteoglycans and fibrous proteins and is a highly glycosylated (and, thus, also a sialylated) environment [39]. Molecular investigations have confirmed that ECM is a physiologically active component of living tissue that provides biochemical and structural support to cells. The highly dynamic structure constantly undergoes remodeling processes, and moreover, each organ is characterized by a unique ECM composition [40]. The dysregulation of ECM dynamics leads to the development of cancer; however, cancer cells are able to modulate the extracellular matrix [40,41,42]. Abnormalities in the ECM also deregulate stromal cell behavior, facilitate angiogenesis, deregulate the activation of immune cells, and promote the formation of a TME [43]. The increased expression of glycoconjugates within the ECM results in worse adhesion to fibronectin and collagen, which influence proliferation, differentiation, and migration, whereas desialylation exerts the opposite effects [44,45]. Furthermore, it has been demonstrated that α-2,6 sialic acid is required for the adhesion of cells to fibronectin, which underlies the adhesion of cancer cells to the ECM [41,45,46,47]. In line with this, sialic acids affect the integrin-mediated intercellular communication between tumor cells and ECM [48,49,50,51]. Furthermore, the sialylated podoplanin (PDPN) on CAFs plays a key role in tumor-cell-induced platelet aggregation; however, the deprivation of sialic acid reduces this function within the TME [52,53,54,55]. The importance of the microenvironment in cancer progression is also based on the interaction of immune cells with tumor cells. Sialic-acid-mediated immunosuppression is known as one of the malignancy-promoting mechanisms (Figure 2C). The upregulation of sialic acid in the tumor microenvironment contributes to the establishment of an immunosuppressive environment through the involvement of immunomodulatory Siglec receptors expressed on tumor-infiltrating immune cells [56] (Figure 3).

Given the high specificity of the distribution of CD33-related Siglecs and differences in the structure of intracellular signaling domains, the interplay between receptors and their ligands determines the activating or suppressive signaling pathways responsible for the function of the immune cells. It is of particular importance in the context of immune homeostasis maintenance. The sialic acid–Siglec axis modulates the balance between self-recognition and non-recognition and mediates cell signaling and adhesion [57,58]. However, inhibitory Siglecs containing an immunoglobulin-like inhibitory motif (ITIM) have been shown to play a role in regulating immune and non-immune responses within the TME and can influence various aspects of cancer progression [59]. In a model of hypersialylated cancer cells, it was shown that such cells behave like a “super-me” and reduce the immune response via binding to Siglec-7 on NK cells [60].

## 3. Drug Resistance in Cancer

### 3.1. Molecular Mechanisms of Drug Resistance in Cancer

Drug resistance is one of the major problems in cancer management. Many drugs are successfully used; however, not fully understanding the molecular profile of cancers does not guarantee a complete cure. Cancer cells develop the ability to resist chemotherapy through many different mechanisms, resulting in no/poor response to therapy. This may be due to the fact that this ability is genetically determined or acquired during the use of chemotherapeutic agents. Multi-drug-resistant (MDR) cancer therapy is a particular challenge, especially in acquired resistance. Tumors of this phenotype are resistant to a wide range of drugs with different structures and mechanisms of action [61]. Although the etiology of MDR is multifactorial, it is mainly associated with the overexpression of energy-dependent transporters (Figure 2D). The main function of these systems is to detect and pump out anticancer drugs, so the overexpression of these ABC family receptors is one of the methods of cancer cell survival. The main member of this family is P-glycoprotein (P-gp), which is able to bind with a wide variety of drugs. This cation pump, a product of the ABCB1 (MDR1) gene, uses ATP to transport hydrophobic substrates [62]. The widespread expression of P-gp in human tissues, including the intestinal epithelium, liver, kidneys, and vascular epithelium, and intracellular distribution suggest its function remains the excretion of endogenous metabolites and the prevention of the entry of toxins, including drugs [63,64,65]. In the field of oncology, P-gp has been extensively studied in different types of cancers, including breast cancer, ovarian cancer, and lung cancer. However, the expression status of P-gp depends on the type of tumor, as it is upregulated in some and downregulated in others [64]. For example, non-small-cell lung cancer (NSCLC) cells after treatment show an increased level of the P-gp gene and a higher level of expression of the P-gp protein compared to cells not treated with a chemotherapeutic drug [66]. Besides the P-gp, the transporter proteins belonging to the ABC family, including ABCC1, ABCB1, BCRP, and LRP, participate in the export of toxic substances and pre-metabolized drugs [67,68]. The MDR of cancer cells may also be related to the intensification of the metabolism and the detoxification processes of cytostatics administered as part of chemotherapy. An example of a molecule responsible for such a resistance mechanism is glutathione s-transferase (GSTP1), characterized by low substrate specificity and high reactivity conditioned by an electrophilic atom [69]. Its increased expression in tumors correlates with the enhanced expression of P-gp and MRP1 at the same time [70,71]. Moreover, drug resistance in cancer can be associated with reduced absorption as a result of the modulation of diffusion across the cell membrane and endocytosis [72]. The MDR is also promoted via extrinsic mechanisms, including insufficient blood flow through tumor structures, which is closely linked with their poor vascularity. This phenomenon occurs in solid cancer with a low degree of vascularity, for which drug availability is limited due to the inhibition transport of molecules into the cells as a result of a decrease in pH that results from hypoxia and the accumulation of lactic acid [73]. It is believed that the increased production of lactic acid, in addition to causing resistance, promotes angiogenesis and metastasis and impedes the function of immune cells [74]. Metabolic activity, ABC transporters’ expression, and the proliferative potential of malignant cells are all modulated via the PI3K/AKT signaling pathway. Its aberrant activation seems to be an important factor in the generation of MDR, as shown in highly chemoresistant cell lines [75]. 

### 3.2. Modulatory Effects of Sialoglycans on Drug Resistance—Related Processes

Scientific reports have suggested that several sialyltransferases, including ST8SIA4, are involved in the activity of the PI3K/AKT pathway [76,77]. ST8SIA4 mediates the activity of phosphoinositide 3-kinase (PI3K)/Akt, and the MDR phenotype positively correlates with the level of this sialyltransferase. Targeting and inhibiting this pathway using a specific inhibitor contributes to mitigating the adverse effects caused by ST8SIA4 overexpression [74]. Additionally, ST6Gal-I also plays a significant role in cell invasion through the PI3K/Akt pathway, as evidenced by the fact that knocking down ST6Gal-I in aggressive prostate cancer cells substantially hindered their growth, proliferation, and migration capabilities by reducing the levels of several pathway components [78]. Sialylation modifications are influenced not only by the overexpression of sialyltransferases but also by sialidases, which are crucial factors. The study by Nath et al. showed that increased membrane-bound Neu2 expression in drug-resistant PDAC cells is linked to intensified apoptosis via Fas activation and weakened PI3K pathway activity, leading to decreased invasiveness, metastasis, and cell proliferation [79]. In addition to the promotion of tumor development, the TME, as a target for sialome machinery, exerts a profound effect on treatment resistance. As shown, the cancer-induced modifications of ECM negatively affect the response to therapy. The high level of density and rigidity of ECM facilitates the engulfment of the cancer cells, creating a “coat” and protecting the cancer cells from treatments. This type of barrier alters the concentration of oxygen, nutrients, and metabolites. Increased levels of hypoxia and metabolic stress, along with increased tissue stiffness, may be associated with chemoresistance [80,81]. Besides the effect of cancer on the ECM structure and composition, CAFs as the largest population of stromal cells mediate the remodeling of the ECM and participate in the development of resistance to anticancer therapy [82,83]. While resistance to cisplatin is well established, the specific resistance to gemcitabine, oxaliplatin, and paclitaxel has been also found to correlate with CAF expression in various cancers [84]. The relationship between immune cells and drug resistance is a complex and extensive area of research. The cellular analysis of TME has revealed the role of tumor-associated macrophages (TAMs) in the growth and metastasis of malignant tissue [85]. Furthermore, it has been shown that the conventional strategies of cancer therapy exert and depend on the tumor-infiltrating macrophages. In the context of reduced drug sensitivity, impaired macrophage-dependent tissue repair processes can result in therapy failure [86]. The investigation by Olson et al. revealed that the cytotoxic effect of paclitaxel is reduced via TAMs [87]. This is consistent with the clinical observation by Yin that the increased infiltration of TAM is associated with chemoresistance in patients with colorectal cancer [88]. This suggests that several populations of immune cells that create TME influence cellular drug sensitivity; however, the knowledge in this field is limited. Finally, the response of malignant cells to therapy may be due to epigenetic modulations. There is increasing evidence that different types of miRNAs are also involved in regulating resistance to anticancer drugs. As shown, downregulated miR-331-5p and miR-27a are associated with resistance to doxorubicin and relapse in leukemia, whereas miR-214 and miR-660-3p are associated with cisplatin resistance in ovarian cancer and gemcitabine resistance in pancreatic ductal adenocarcinoma, respectively [89,90,91].

## 4. Sialic Acids as Targets and Cellular Indicators of Drug Action

### 4.1. Effects of Chemical Compounds on Sialome Machinery

There are several groups of drugs that have been shown to affect sialylation either by altering the expression of enzymes involved in sialylation or by directly affecting the sialylated glycoconjugates. Currently, there are no drugs that have been specifically designed to change sialylation, as these processes are complex and not fully understood. Some drugs were developed and approved for well-defined therapeutic indications, and their effects on sialic acid or the expression of sialidases or sialyltransferases may be an unintended consequence of their primary mechanism of action. Several drugs have been developed to inhibit sialidases with the goal of blocking the cleavage of sialic acid residues from glycans. Sialidase, as an enzyme associated with influenza viruses, is responsible for the breakdown of sialic acid on the surface of the cell membrane and facilitates the entry of a virus into the cell [92]. Primarily, the sialidase inhibitors, including oseltamivir and zanamivir, demonstrate significant clinical benefit and are widely used for the post-exposure prophylaxis of seasonal influenza. Interestingly, there is an increasing body of evidence that the biochemical targeting isoenzymes of human sialidases can be useful in sialidase-mediated pathological conditions [93,94]. Indeed, the role of sialidases has been confirmed in pathological cardiac hypertrophy, whereas the inhibition of Neu1 activity via oseltamivir and zanamivir exerts a cardioprotective effect [95]. Conversely, the oseltamivir-induced alterations in the sialylation pattern and the impairment of sialidase activities in canine mammary tumors were correlated with the enhanced aggressiveness of malignant cells [96]. Given the potential role of hypersialylation in tumorigenesis, the impairment of sialome-promoting machinery can be a promising direction in the development of anticancer therapies. Both a fluorinated sialic acid analog and carbamate-based analogs have been used to interfere with hypersialylation, block the sialic acid recognition pathway, and lead to a reduction of cell motility and invasion [97,98,99,100,101].

### 4.2. Molecular and Cellular Consequences of Sialome Targeting

The glycosylation pattern is a specific feature of the molecular modification of membrane glycocalyx in various cancers. Among the human gangliosides, unusual sialylation has been described in cancer cells via the attachment of N-glycolylated sialic acid (NeuGc) to ganglioside GM3 [102]. Interestingly, a change from N-glycolyl- to N-acetyl-sialic acid in the GM3 ganglioside reduces tumor development in mouse leukemia cells [103]. These unique features of N-glycolyl GM3 in several tumors suggest its usefulness in immune-based diagnosis and therapy [104]. In this field, therapeutic strategies and several immunotherapeutic approaches have emerged, including combination therapies with monoclonal antibodies. Racotumomab is an anti-idiotype antibody capable of inducing anti-N-glycolyl-GM3 antibodies in neuroblastoma patients, as investigated in a multicenter clinical trial (Clinical Trial NCT02998983). Enhanced sialylated N-glycolyl GM3 expression has been found in neuroblastoma and described as a useful specific target in immunotherapy. Racotumumab is also now used as second-line therapy in non-small-cell lung cancer and prolongs the overall survival of patients [105,106]. 

**Figure 4:**
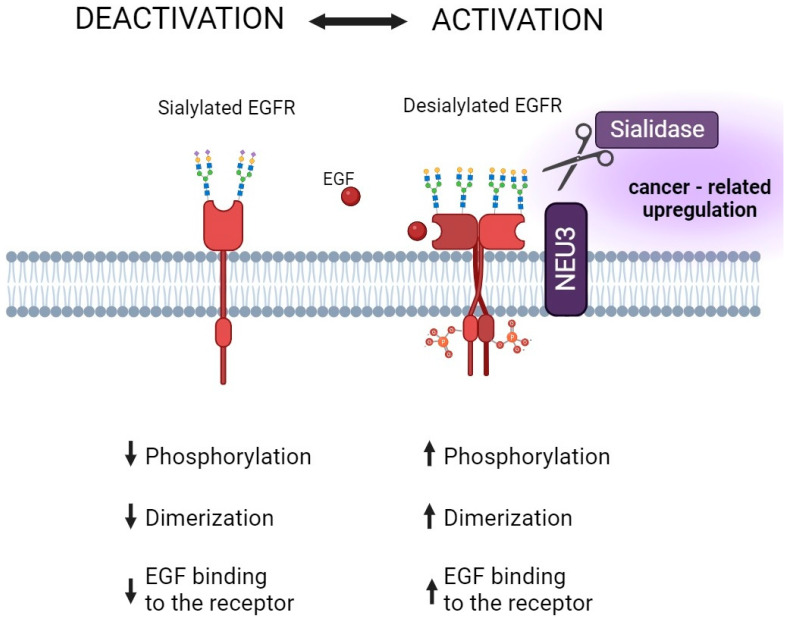
Effects of sialylation and desialylation on EGFR functionality. In cancer cells, an increase in the expression of membrane-bound sialidase NEU3 was observed, strongly correlating with desialylation and the activation of the EGFR receptor. This intricate process intensifies receptor phosphorylation and dimerization and enhances its affinity for EGF binding. Additionally, experimental treatment of cell surfaces with sialidase significantly enhances EGFR activation. In the context of cancer, a significant upregulation of NEU3 and other neuraminidases has been observed, confirming their key role in its pathogenesis.

The new therapeutic approach targets the hypersialylated surface of cancer cells or Siglec-7 and Siglec-9 receptors that bind sialic acid. This action is aimed at enhancing the response of natural killer cells since it is assumed that the sialic acid–Siglec axis may downregulate cytotoxicity [107]. Sialidase antibody conjugates may enable the targeted destruction of sialylglycans to enhance the anti-tumor immune response [60]. Additionally, the different subtypes of sialyltransferase inhibitors represent an attractive group of drugs with high potential for clinical use in cancer. There are many ideas and much research on agents affecting sialyltransferases, but the pace of their design and synthesis is slow. This is due, for example, to the fact that the strongest nucleotide-based inhibitors are characterized by poor penetration through the cell membrane [108]. Development is also hampered by the paucity of in-depth biological research into the mechanisms of action of sialyltransferases. Although the primary mechanisms of action of the most routinely used drugs are not directed at the sialome, the modification of glycocalyx can be considered a molecular detail of not fully understood importance. In addition to the cytotoxic effects of antimetabolites, alterations in the cell membrane sialylation pattern were also observed. In 1985, researchers became interested in whether drugs that affect the intracellular cytidine triphosphate (CTP) pool, an important nucleotide intermediate in sialylation reactions, would also affect the level of sialic acid expressed on cell membranes. As shown, several medications, such as 3-deazauridine, acivicin, 1-β-d-arabinofuranosylcytosine, and hydroxyurea, alter the turnover of sialic acid and its de novo synthesis, whereas doxorubicin has no effect on either the synthesis or expression of sialic acid on the cell membrane [109]. According to the authors, the drug-induced inhibition of sialylation should be further investigated, as it may modify cell biology and contribute to anti-cancer activity. Glycosylation disturbances have been detected in patients treated with methotrexate (MTX) for rheumatoid arthritis (RA). The altered glycosylation of IgG was strongly connected with pathogenesis and changes in the course of the disease [110]. It is of particular importance since it is widely known that altered sialylation in RA contributes to the regulation of arthritogenicity [111]. Lundström et al. have suggested that changes in the glycome of serum can be considered potential indicators of MTX clinical response prediction [112]. Molecular studies have revealed that the glycosylation status of growth factor receptors determines signal transduction pathways in malignant cells and thereby modulates cell growth and proliferation. In the case of the epidermal growth factor (EGF) receptor, its activation and dimerization depend on sialidase-1 (Neu-1) and sialidase-3 (Neu-3) activity resulting in tumorigenesis [28,29,113] (Figure 4).

A study by Qorri et al. demonstrated that aspirin and celecoxib dampen Neu-1 activity in pancreatic cancer cells, inducing apoptosis and necrosis in a dose- and time-dependent manner. This finding may be of significant importance, as it may be the missing link between the anticancer efficacy of NSAIDs and the role of glycosylation in inflammation and sialic acidgenesis [113]. Additionally, the therapeutic targeting of Neu-1 with oseltamivir and aspirin with gemcitabine (GEM) treatment significantly disrupts critical signaling mechanisms, tumor progression, and metastasis in a preclinical mouse model of human pancreatic cancer [114]. The enhanced level of sialic acid was detected in the serum of rosiglitazone-treated diabetic patients and significantly correlated with cardiovascular risk factors. This observation indicates the importance of the sialylation pattern as an indicator of possible drug-induced negative effects [115].

## 5. Sialic Acids as Modulators of Cellular Drug Sensitivity in Cancer

### 5.1. Sialylation and the Efficacy of Protein Kinase Inhibitors in Cancer Therapy

As mentioned in previous sections, glycocalyx is an integral part of the cell membrane. Glycan chains associated with proteins and lipids support the malignant cells’ survival via a direct impact on cell growth and survival [116]. The high flexibility of sugar structures is associated with the enzymes of glycome-modifying machinery that present different expressions in both normal and malignant cells [117]. There is increasing evidence that a hypersialylated tumor cell surface promotes drug resistance, but the exact mechanism of this phenomenon is unclear. Given the chemical and physical properties of sialic acids and their modulatory influence on protein function and structure, membrane sialoglycans can the regulate cellular sensitivity to drugs through the direct influence on drug activity, the drug’s reduced ability to bind to hypersialylated cancer cells, and the modification of the function of proteins related to cell division. However, evidence has been provided that aberrant sialylation, and especially sialyltransferases overexpression, and the abnormal (usually decreased) expression of sialidases contribute to therapy resistance in cancer [118]. Changes in the sialylation pattern have been detected in cells and tissues exposed to various stimulatory agents, including ethanol, heavy metal stress hormones, and narcotic substances as a result of an altered balance between activities and the expression sialyltransferases and sialidases [119,120,121,122,123]. In the field of cancer treatment, radiation exposure was discovered to enhance the sialylation of membrane glycoproteins and contribute to cellular radiation resistance. Lee et al. observed that both human lymphoma B-cells and leukemic monocytes express a sialylated form of integrin β1 in response to γ-rays exposure [117]. These changes were associated with the increased expression of β-galactoside α(2,6)-sialyltransferase (ST6Gal-1), known as the primary enzyme engaged in the α2-6 sialylation of glycoproteins. The increased survival following radiation exposure in analyzed cells was related to the enhanced structural stability of membrane sialylated glycoproteins and decreased radiation-induced cell death and caspase 3 activation. These effects were not observed in sialidase-2-treated cells or in response to the knockdown of expression via short interfering RNA targeting ST6Gal-1 [117]. Interestingly, an investigation by Smithson et al. revealed that radiation-induced ST6Gal-1 affects the function of pro-apoptotic proteins, including tumor necrosis factor receptor 1 (TNFR1) and the Fas receptor in rectal adenocarcinoma, resulting in the decreased propagation of apoptotic signaling [124]. Therefore, ST6Gal-1 is considered a biomarker of ionizing radiation and related therapeutic resistance in various cancers. Numerous studies have demonstrated that the chemoresistance corresponds to the ST6Gal-1-mediated functional alterations in receptor proteins and downstream signaling responsible for cell proliferation and tumorigenesis. Park et al. found that ST6Gal-1 induces EGFR sialylation in human colon cancer cells. Based on this finding, the regulatory function of sialic acid on the cytotoxic effect of EGFR kinase inhibitors was described. As shown, the sialylation of the EGFR affects EGFR-mediated cell growth and reduces sensitivity to gefitinib in human colon cancer cells, whereas the anticancer effect of gefitinib was augmented in ST6Gal-1-deficient cells [125]. Similar effects were described by Britain et al. in the OV4 ovarian cancer cell line and confirmed that the sialylation of EGFR correlates with ST6Gal-I expression and protects against gefitinib-mediated apoptosis [126]. In another study, it was concluded that cellular sialylation can regulate EGFR phosphorylation by modulating the activity of other kinases responsible for EGFR phosphorylation, as well as directly suppressing EGFR autophosphorylation. Sialylation may partially suppress EGFR phosphorylation and increase EGFR sensitivity to tyrosine kinase inhibitors (TKI). The data from the study suggest that site-specific EGFR phosphorylation plays an important role in the maintenance of TKI resistance and that targeting these selective EGFR phosphorylations may be a future direction for drug discovery [127]. In addition to EGFR, the regulatory function of ST6Gal-1 in human epidermal growth factor receptor 2 (HER2)-targeted therapy was described in human gastric cancer. A study by Liu et al. revealed that ST6Gal1 overexpression induced high levels of HER2 sialylation and increased cell viability. As a consequence, the α2,6-sialylation of the receptor provided trastuzumab-mediated protection against apoptosis. These effects were accompanied by potentiated cell cycle arrest in the G2/S phase, decreased caspase-3 levels, and enhanced Akt and ERK phosphorylation levels [128]. Similarly, the post-translational modification of fibroblast growth factor receptors (FGFR) via ST6Gal-1 promotes cancer progression and malignancy and underlies the drug resistance in epithelial ovarian cancer. Among the FGFR family, the α2,6-sialylation of FGFR1 enhanced the expression of phospho-ERK1/2 and phospho-focal adhesion kinase, thus promoting cell proliferation. 

### 5.2. Sialylation and the Cellular Sensitivity to Cytotoxic Drugs

According to Ou et al., the overexpression of ST6Gal-1 attenuates the sensitivity of ovarian cancer cells to several anticancer drugs, including paclitaxel, Adriamycin, and the FGFR1 inhibitor, signed as PD173074 [129]. Although ST6Gal-1 is known as the main player in sialic-acid-related cancer progression and therapeutic resistance, sialyltransferase ST3Gal1 has also been linked to cancer progression and drug resistance. The overexpression of ST3GAL1 was found to increase paclitaxel resistance in ovarian cancer, while the downregulation of ST3GAL1 reduced paclitaxel resistance in vitro in cells, as well as reducing the therapeutic effect of paclitaxel on tumor growth in mice [130]. Additionally, a high level of ST3Gal III contributes to taxol resistance in ovarian cancer, rendering the therapy less effective [131]. The sialotransferase-induced chemoresistance to cisplatin was found in numerous ovarian cancer cell lines by Schultz et al. [132]. The authors suggested that the precise mechanism of this phenomenon is not yet understood; however, the relationship between ras-dependent cellular resistance to cisplatin and the ras-signaling-related regulation of ST6Gal-1 is part of the multifactorial regulatory pathway of cellular sensitivity. An interesting finding related to sialic acid and its effect on treatment is the dependence between the therapeutic effect of drug action and the expression of sialyl Lewis-X (sLx) and sialyl Lewis-A (sLa) epitopes on cancer cells. A study by Matsumoto et al. showed that the beneficial effect of cimetidine co-administered with 5-FU after colorectal cancer surgery depended on the degree of expression of the sLx and sLa epitopes on cancer cells [133]. Cimetidine treatment significantly reduced the frequency of metastases and significantly increased the survival rate of patients whose cancer cells expressed higher levels of sLx and sLa epitopes but not patients whose cancer cells expressed no or lower levels of these epitopes, although such cancers are considered less aggressive [133]. Functional analysis of the sialylation pattern in cholangiocarcinoma cells (CCA) revealed that both α2,3- and α2,6-sialylation participate in chemoresistance to 5-fluorouracil, whereas the suppression of sialylation reverses this process. Wattanavises et al. speculated that the pan-sialylation inhibitor 3Fax-peracetyl-Neu5Ac can be a useful candidate for chemosensitization in the course of CCA therapeutic management [134]. Besides the biochemical inhibition of sialylation with fluorinated sialic acid derivatives, there is evidence linking miRNAs to the level of sialyltransferase expression and chemoresistance, as mentioned previously. The investigation by Li et al. confirmed the importance of MiR-4701-5p as a negative regulator of ST3GAL1. The transfection of highly resistant chronic myeloid leukemia cells with MiR-4701-5p directly targeted ST3GAL1 to reduce resistance to adriamycin, paclitaxel, and vincristine [135]. There is increasing evidence that several miRNAs, including miR-195-3p, miR-181c, miR135b, and miR-182, target sialyltransferases’ expression and convert resistant cells to susceptible cells [136,137,138]. The targeting of ST8Sia4 with miR-181c inhibited its expression and sensitized malignant cells to adriamycin via the phosphoinositide-3 kinase (PI3K)/AKT signal pathway [137].

### 5.3. Sialidases as Therapeutic Target to Counter Chemoresistance

Since it is well established that the presence of upregulated sialic acid on the surface of cancer cells correlates with metastasis, disease progression, and resistance to chemotherapy, neuraminidase has garnered significant attention as a promising therapeutic target to counter chemotherapy resistance [139]. It was shown that the utilization of the viral neuraminidase inhibitor oseltamivir demonstrated the ability to overcome chemoresistance in a dose-dependent manner [140]. The targeting of sialidase-1 with oseltamivir disables cancer cell survival in human pancreatic cancer with acquired chemoresistance to cisplatin and gemcitabine. Furthermore, oseltamivir phosphate was observed to reverse the epithelial–mesenchymal transition, known as a critical process implicated in cancer metastasis. These findings underscore the synergistic potential of oseltamivir in conjunction with anticancer drugs, highlighting its impact on enhancing the viability of chemoresistant cells [140]. While the therapeutic mechanisms of oseltamivir may involve multiple molecular pathways, this finding provides compelling evidence that neuraminidase inhibitors hold promise as potential agents to counter chemoresistance in cancer. The clinical observations indicate a potential inhibitory effect of oseltamivir not only on viral neuraminidase but also on endogenous human sialidases [141]. Drawing from the paradigm set by oseltamivir, which enhances the efficacy of anticancer drugs when administered concomitantly, an inquiry has arisen concerning the existence of other substances that exhibit analogous effects while manifesting minimal side effects. The exploration of such compounds has assumed paramount significance, particularly in light of the potential occurrence of adverse effects, as observed in the case of oseltamivir [142]. In addition to synthetic compounds that modulate sialic acid levels, natural compounds of plant origin have also garnered attention. Among them, honokiol (HNK) has been extensively investigated in numerous studies. HNK is a lignan extracted from the *Magnolia grandiflora* traditionally employed in inflammation treatment. As a bioactive agent, HNK exhibits a diverse range of biological activities and engages with several molecular targets that result in antiviral, anti-inflammatory, antioxidant, and antiangiogenic activities [143,144,145,146]. However, its most noteworthy attribute is its potential as a potent anticancer agent distinguished via its remarkable safety profile and low toxicity [147]. Additional observations have indicated that HNK has the ability to regulate the expression of sialidase-1. By inhibiting sialidase-1, HNK reduces the level of sialic acid, as confirmed in breast cancer cell lines [148]. Furthermore, this compound has been investigated in other cancer cell lines, such as glioma cells, when used in combination with temozolomide and in oral squamous cell carcinoma (OSCC) cells in conjunction with 5-FU [149,150]. In both cases, HNK enhanced the proapoptotic effects of these drugs. It is also worth noting that HNK improved the efficacy of doxorubicin (DOX) in breast cancer, thereby reducing its toxic effects [151]. Furthermore, in the liposomal form, honokiol may be a new potential treatment option for patients with recurrent glioblastoma [152]. The significant potential in cancer therapies has been described in the case of an herbaceous plant *Sanguisorba officinalis*. Its co-administration with 5-fluorouracil (5-FU), a first-line drug used in colorectal cancer (CRC) known for its susceptibility to drug resistance, has been demonstrated to synergistically enhance cytotoxicity against cancer cells [153]. Intriguingly, *Sanguisorba officinalis* also manifests neuraminidase inhibitory activity, further substantiating its potential as an adjunct therapeutic agent with inherent anticancer activity [154,155]. Another botanical resource exhibiting potent therapeutic potential is *Ginkgo biloba* due to its anti-inflammatory, antibacterial, and cardiovascular effects. Notably, scientific evidence indicates that a bioactive bioflavonoid compound extracted from Ginkgo biloba leaves, known as ginkgetin, possesses anticancer properties [156,157]. Combinatorial treatment involving ginkgetin has been found to enhance the therapeutic efficacy of cisplatin in non-small-cell lung cancer (NSCLC) [158]. These effects are related to unbalanced redox hemostasis in cisplatin-treated cells; however, the multiple mechanisms of action are attributed to ginkgetin, including its activity against sialidases [159,160]. This aspect renders it a potential agent for targeting sialic acids, possibly applicable to chemoresistant cancers, although research in this area remains limited. Further exploration of this avenue could prove valuable in developing novel therapeutic strategies for combating drug-resistant cancer types. The potent inhibitory properties against sialidase have been described in in vitro investigations that have identified seven compounds, notably crocin, picrocrocin, safranal, rutin, and apigenin, isolated from saffron (*Crocus sativus*). These research findings have revealed the extracts’ robust cytotoxic activity against melanoma cells (IGR39), triple-negative breast cancer (MDA-MB-231), and glioblastoma cell lines (U-87) and indicated the potential future utility of saffron in the realm of anticancer therapy [161]. A substantial number of herbal substances, particularly those employed in Chinese traditional medicine, such as *Amomum villosum*, *Melaphis chinensis*, and *Flos Caryophylli*, exhibit robust neuraminidase inhibitory activity comparable to that of oseltamivir [154,162]. Additionally, various other herbs and formulations whose precise pharmacological activities are yet to be fully elucidated, including *Lonicerae Japonicae Flos*, *Yupingfeng San*, and *Huanglian Jiedu Decoction*, have been shown to exert inhibitory effects on human neuraminidases [163]. Consequently, the investigation of natural compounds with neuraminidase and/or sialyltransferase modulatory activity presents an unexplored avenue with significant potential to augment existing cancer treatments and combat chemoresistance [164,165,166,167,168,169,170].

### 5.4. Sialic-Acid-Rich Glycocalyx and Chemoresistance in Brain Tumor Therapy

The discovery of both synthetic and natural substances related to sialome-related therapeutic efficiency is of particular importance in the field of therapy of malignancies of high resistance status. An extremely interesting issue is the role of sialic acids in drug resistance in brain tumors. Of particular interest in this context are gangliosides, which are the dominant sialic acid carriers in the brain. While the concentration of sialic acid can vary, depending on the specific tissue; there is evidence to suggest that the brain contains a relatively high level of sialic acid compared to other organs [171,172]. Furthermore, ganglioside structures are more complex in the brain than in tissues [173]. A structural analysis by Mikami identified a broad expression of several gangliosides in the brain, including GM4, GM3, GM2, GM1, GD1a, GD1b, and GT1b, that predominantly possess N-acetyl neuraminic acid; however, a minor amount of N-glycolyl neuraminic acid was also described [174]. It is of particular importance in the context of the role of sialylated glycolipids in brain malignancy. The abundant and complex expression of gangliosides in the brain may implicate brain-specific biological functions that are also present in the process of carcinogenesis and chemoresistance. An investigation by Fabris et al. revealed changes in the expression of gangliosides with a higher abundance of simple structures in human gliomas [175]. Besides their function as tumor-associated antigens, the low-sialylated gangliosides, including GM3, GD2, and GD3, have been described as the crucial promoters of invasive potential and aggressiveness in glioblastoma and correlate with the degree of tumor malignancy. This may suggest that mono- and disialogangliosides are attractive molecular targets for brain tumor therapy [176]. Interestingly, the alterations in glycosphingolipid metabolism are beneficial in the course of lysosomal storage disorders in the central nervous system. There is evidence that miglustat and eliglustat effectively inhibit the proliferation of primary diffuse midline glioma cells. Although the mechanism of this phenomenon is not directly related to the sialylation pattern, the inhibition of glucosylceramide formation seems to be beneficial in glycolipid-related pathologies. Additionally, glucosylceramide synthase inhibitors induced ceramide accumulation in glioma cells and synthesized the malignant cells with irradiation and temozolomide [177]. The main uncertainties related to the chemoresistance of brain tumors include the exact mechanism of this phenomenon, its relationship to tumor heterogeneity, and the identification of biomarkers. High aggressiveness and histological heterogeneity and molecular diversity underlie the low clinical efficacy of the standard therapy with temozolomide [178,179]. The concomitant administration of temozolomide and dexamethasone is the basic pharmacological management of limited efficiency in malignant brain tumors. There is increasing evidence that dexamethasone (Dex) affects the sialylation pattern and can reduce patient overall survival (OS) and progression-free survival (PFS); however, the role of sialome-related changes in low therapeutic susceptibility was not confirmed. Interestingly, sialic-acid-mediated immunosuppression can be considered a poor prognostic factor in glioblastoma patients [180]. The influence of drugs and potential therapeutic agents on sialome-related machinery and their importance in cancer management, including chemoresistance, are presented in Table 1 and Table 2.

## 6. Conclusions

Sialic acids are ubiquitous molecules, and their particular importance is still being discovered in the cancer process and the influence on drug resistance. The study of the molecular mechanisms in which sialic acid is involved is the basis for the development of effective drugs and new therapeutic methods. The complexity of the topic and the lack of a full explanation of how sialic acid works can be a cause for concern, but progress in this field gives hope. Discovering hitherto unknown mechanisms of known drugs opens the door to their new applications, especially to drug-resistant cancers. The most promising therapeutic agents that are still being designed and synthesized include sialyltransferase inhibitors, antibodies and inhibitors targeting Siglecs and selectins, and sialidase antibody conjugates. An interdisciplinary effort is required to decipher the characteristics and biological effects of the altered sialome in order to put this knowledge into practice for the benefit of cancer patients.

## Figures and Tables

**Table 1:** The importance of sialidase inhibitors in cancer management.

Drug		Target	Biochemical Effect	Other Effects	Cancer Type
Neuraminidase Inhibitors	Oseltamivir Zanamivir	Inhibit: ►Virus neuraminidase ►Human sialidase NEU1 NEU2	Altered sialylation pattern	Reversal of EMT; increased drug sensitivity	Lung cancer (oseltamivir) [166]
	Honokiol (HNK)	Regulate the expression of Neu1 Suppress Neu1 protein expression		Anti-tumor, anti-inflammatory, antioxidant, and antiangiogenic activity, enhancing the proapoptotic effects of drugs	Breast cancer (MCF, MDA) [143,144,145,146,147,148,149,150,151,152]
	*Sanguisorba officinalis*	Neuraminidase activity		*Sanguisorba officinalis +* 5FU → synergistically enhancing cytotoxicity	Colorectal cancer [153]
	*Ginkgo biloba* (ginkgetin)	Viral sialidases		Anti-tumor activity, inhibited tumor growth, enhancing chemotherapy sensitivity, and reversing chemoresistance	Ovarian cancer cell lines, prostate cancer cells (DU-145), and gastric cancer cells [156,157,158,159,160,165]
	*Crocus sativus*	Neuraminidase		Cytotoxic activity on cancer cells	Melanoma cells (IGR39), triple-negative breast cancer(MDA-MB-231), and glioblastoma cell lines (U-87) [161]
	*Amomum villosum, Melaphis chinensis (Galla Chinensis), Caryophylli Flos, Lonicera Japonica, Yupingfeng San, Huanglian Jiedu*	Neuraminidase and probably others		Inhibitory effects on tumor growth, synergizing the antitumor effect of DDP (*Lonicera Japonica*)	Various types of cancer, gastric cancer, and lung cancer [166,167]

**Table 2:** Effects of drugs and potential therapeutic agents in sialome-related machinery and their importance in cancer management.

Drug		Target	Biochemical Effect	Other Effects	Cancer Type
Antimetabolites	3-dezauridine (competitive inhibitor of CTP synthetase)	Limiting resialylation without affecting de novo synthesis	Decreased sialic acid expression on cell surface	Diverse and substance-dependent	Various types of cancers [109]
	Acivicin	Limiting resialylation and de novo sialic acid synthesis delay			
	1-beta-D-Arabinofuranosylcytosine	Inhibited both de novo sialic acid synthesis and membrane resialylation			
Racotumomab		NGc-containing gangliosides trigger the immune system against tumor antigen NGcGM3(mimics NGc gangliosides)	Decrease in sialic acid levels	Additive antitumor effect	Non-small-cell lung cancer model [105,106]
Aspirin and celecoxib		Inhibit Neu-1 activity	Decrease in sialic acid levels	Inducing apoptosis and necrosis	Pancreatic cancer cells [113,114]
Metformin and rosiglitazone		N/A	Higher serum sialic acid concentrations	Anti-tumor activity	Prostate cancer model [168]
Soyasaponin I		ST3Gal-IV	Depress mRNA expression of ST3Gal-IV and attenuate α2,3-sialylation on the cell surface	Inhibit the migration ability of cancer cells, enhance cell adhesion to extracellular matrix proteins, and reduce metastasis	Breast cancer cells and lung cancer (mice) [108,181]
Lithocholic acid and its derivatives		α2,3-sialyltransferase (ST3Gal-I)	↓ sialic acid levels	Induces apoptosis and reduces cancer cell proliferation, aggressiveness, and the metastatic potential of primary tumors	Glioblastoma [168], Ewing sarcoma [169] and breast cancer [168,169,170,171]
Transition-state analogs		Sialyltransferase, mimics the substrate CMP-Neu5Ac	The highest inhibitory activity and lower level of sialic acid expression on the cell surface	Potential new antimetastatic agents	Various types of cancers [182]

## Data Availability

Not applicable.
